# Evaluation of the usefulness of costovertebral angle tenderness in patients with suspected ureteral stone

**DOI:** 10.1002/jgf2.581

**Published:** 2022-09-12

**Authors:** Hiroyasu Higuchi, Taku Harada, Juichi Hiroshige

**Affiliations:** ^1^ Division of General Medicine Showa University Koto Toyosu Hospital Tokyo Japan; ^2^ Division of Diagnostic and Generalist Medicine Dokkyo Medical University Hospital Tochigi Japan

**Keywords:** costovertebral angle tenderness, physical examination, ureteral stone

## Abstract

**Background:**

The usefulness of costovertebral angle (CVA) tenderness for the diagnosis of a suspected ureteral stone remains controversial.

**Methods:**

This single‐center, retrospective, observational study included patients aged 16–64 years with acute‐onset unilateral lower back pain or abdominal pain. The diagnostic accuracy of CVA tenderness was investigated.

**Results:**

In total, 132 patients met the criteria; 80 were diagnosed with ureteral stones. The sensitivity and specificity of CVA tenderness were 0.65 and 0.50, respectively; positive and negative likelihood ratios were 1.3 and 0.7, respectively.

**Conclusions:**

CVA tenderness cannot be used as a single diagnostic indicator to confirm or exclude ureteral stone diagnosis.

## Abstract 2

The usefulness of costovertebral angle (CVA) tenderness in patients with suspected ureteral stones remains controversial. CVA tenderness does not contribute to the diagnosis of ureteral stones in this prospective observational study.

## 1 INTRODUCTION

Ureteral stone is one of the most common diseases in primary care,^1^ and accurate timely diagnosis is considered important. Diagnosis is based on medical history, physical examination, urinalysis, ultrasonography, kidney–ureter‐bladder radiography, and computed tomography (CT). However, although plain film radiography is useful for determining the course of known stones, it is not useful for diagnosis in the acute phase.^2^ Noncontrast CT is recommended for first‐episode ureteral stone,^2^ and pretest probability should be increased based on history, physical examination, and simple tests to avoid CT scan overuse.

When a ureteral stone is suspected in a patient with acute onset unilateral abdominal or back pain, costovertebral angle (CVA) tenderness is often noted.^3^ Eskelinen et al. reported that lateral loin tenderness has a sensitivity of 15%, specificity of 99%, and LR+ 30.^3^ On this basis, Japanese guidelines for acute abdomen recommend CVA tenderness for the diagnostic clue of ureteral stones.^4^ However, Moore et al. reported a cohort odds ratio of 0.4 for lumbar or back tenderness for ureteral stones,^5^ while Kartal et al. reported a diagnostic odds ratio of 2.0 for CVA tenderness.^6^ Additionally, CVA tenderness is associated with several diseases.^3^, ^6^ Therefore, the usefulness of CVA tenderness for ureteral stone diagnosis is controversial. Accordingly, we evaluated its usefulness for diagnosing ureteral stone in this study.

## 2 METHODS

This single‐center retrospective observational study was based in a 400‐bed acute‐care university hospital in an urban area. It was conducted over 12 months, from April 1, 2020, to March 31, 2021. Inclusion criteria were patients aged 16–64 years who visited the emergency department for acute‐onset unilateral back pain or abdominal pain. CT imaging was performed in all cases where the examining physician judged that the pretest probability of ureteral stone was high, whereas the decision to perform CT imaging when the pretest probability of ureteral stone suspicion was low was made by the physician responsible for each case. The reference standard of ureteral stone was the presence of a ureteral stone at a site concordant to the symptoms on CT in a patient with acute‐onset unilateral abdominal or low back pain. Exclusion criteria were fever over 37.5°C, diarrhea, trauma, pregnancy, and no documented CVA tenderness.

Age, gender, clinical course, CVA tenderness, and diagnosis were investigated. To test for CVA tenderness, the examiner placed one hand over the region inside the CVA and tapped that hand gently with the closed first of the other hand. Tapping intensity was at the discretion of each physician. Positive CVA tenderness was defined as an exacerbation of pain and a left–right difference. Positive and negative likelihood ratios (LR+, LR‐) were calculated for usefulness of CVA tenderness. Statistical analyses were performed using EZR (Easy R) software^7^ and was calculated using the chi‐square test.

This research was approved by the ethical review board of Showa university and conducted according to the Declaration of Helsinki. The requirement for written informed consent was waived by the ethical review board of Showa University owing to the retrospective design. An opt‐out method was used so that patients could refuse to participate in the study. The collected data was converted to deidentified data.

## 3 RESULT

Of 4249 patients who visited the emergency department, 132 met the inclusion criteria and were included. Ninety‐nine patients (75%) were men, with a median age of 46 (37–53) years, and 80 (60.1%) were diagnosed with ureteral stone. Diagnoses of the 52 patients without ureteral stones were as follows: undiagnosed (*n* = 20), acute appendicitis (*n* = 5), constipation (*n* = 5), acute back pain (*n* = 3), peristaltic pain (*n* = 3), acute cholecystitis (*n* = 2), ovarian tumor torsion (*n* = 2), acute pancreatitis (*n* = 2), cholelithiasis (*n* = 1), acute cholangitis (*n* = 1), pneumonia (*n* = 1), ischemic colitis (*n* = 1), adnexitis (*n* = 1), ovarian tumor (*n* = 1), acute diverticulitis (*n* = 1), ruptured liver tumor (*n* = 1), renal infarction (*n* = 1), and small bowel obstruction (*n* = 1).

Of the 80 patients with ureteral stones, 52 were positive for CVA tenderness. Of the 52 patients without ureteral stones, 26 were positive for CVA tenderness. The CVA tenderness results are shown in Table 1, and the diagnoses are listed in Table 2. The sensitivity of CVA tenderness for ureteral stone was 0.65 (95% confidence interval [CI]: 0.58–0.72), specificity was 0.5 (95% CI: 0.40–0.60), positive likelihood ratio was 1.3 (95% CI: 0.97–1.79), and negative likelihood ratio was 0.7 (95% CI: 0.48–1.05). The chi‐square test showed no significant difference between the two groups with positive and negative CVA tenderness (*p* = 0.10).

**TABLE 1:** Results of costovertebral angle tenderness

	Positive	Negative
Ureteral stone	52	28
Other disease	26	26

**TABLE 2:** Diagnoses of the patients with each group

Diagnosis	Costovertebral angle Tenderness(+)	Costovertebral angle Tenderness(−)	Total
Ureteral stone	52	28	80
Undiagnosed	7	13	19
Acute appendicitis	2	3	5
Constipation	5	0	5
Acute back pain	3	0	3
Peristaltic pain	1	2	3
Acute cholecystitis	1	1	2
Ovarian tumor torsion	1	1	2
Acute pancreatitis	1	1	2
Choletithiasis	1	0	1
Acute cholangitis	1	0	1
Pneumonia	0	1	1
Ischemic colitis	1	0	1
Adnexitis	0	1	1
Ovarian tumor	0	1	1
Acute diverticulitis	0	1	1
Ruptured liver tumor	0	1	1
Renal infarction	1	0	1
Small bowel obstruction	1	0	1
Total	78	54	132

## 4 DISCUSSION

In this study, we found that CVA tenderness cannot be used as a single diagnostic indicator to confirm or exclude ureteral stone diagnosis. Previous studies have examined the usefulness of CVA tenderness and back tenderness in ureteral stone diagnosis.^3^, ^5^, ^6^ Eskelinen et al. found that the sensitivity and specificity of lateral loin tenderness were 0.15 and 0.99, respectively, but the prevalence of ureteral stones in the overall population was very low, about 4%.^3^ Moore et al. reported a ureteral stone prevalence of approximately 50–60%, with a cohort OR of 0.4 (95% CI: 0.2–0.6) for lumbar or back tenderness.^5^ Kartal et al. recorded a ureteral stone prevalence of 77%, and a diagnostic OR of 2.07 for CVA tenderness (95% CI: 0.915–4.660).^6^ Our finding that CVA tenderness is not useful during physical examination for suspected ureteral stone in settings with a high prevalence of ureteral stone is largely consistent with previous studies. In both the present and previous studies, CVA tenderness was positive in several diseases other than ureteral stone. Therefore, CVA tenderness should not be used as a single diagnostic indicator, but rather to clarify the localization of the patient's chief complaint of pain or assist in clinical reasoning.

The limitations of this study include the following: the setting was an emergency room, not a general outpatient clinic; as this was a single‐center emergency room, the study does not have external validity; reproducibility was not assessed using the kappa coefficient; elderly patients with low prevalence and low diagnostic accuracy on physical examination were excluded; and the failure to investigate characteristics of patients with negative CVA tenderness or positive CVA tenderness in nonureteral stone patients.

In conclusion, in patients presenting to the emergency department with acute‐onset unilateral abdominal or low back pain, the presence of CVA tenderness does not contribute significantly to the diagnosis of ureteral stone.

## CONFLICT OF INTEREST

The authors have stated explicitly that there are no conflicts of interest in connection with this article.
